# Do stroke clinical practice guideline recommendations for the intervention of thickened liquids for aspiration support evidence based decision making? A systematic review and narrative synthesis

**DOI:** 10.1111/jep.13372

**Published:** 2020-02-21

**Authors:** Arlene McCurtin, Pauline Boland, Maeve Kavanagh, Dominika Lisiecka, Caoimhe Roche, Rose Galvin

**Affiliations:** ^1^ School of Allied Health, Health Sciences Building University of Limerick Limerick Ireland; ^2^ Health Research Institute University of Limerick Limerick Ireland; ^3^ School of Nursing and Midwifery University College Cork Cork Ireland; ^4^ Department of Nursing and Healthcare Sciences Institute of Technology Tralee Ireland

**Keywords:** clinical guidelines, evidence‐based medicine, medical informatics, systematic reviews

## Abstract

**Rationale:**

Aspiration is a common sequela post stroke as a result of oropharyngeal dysphagia. It is primarily managed using the poorly empirically supported intervention of thickened liquids. Where evidence is limited, clinicians may rely on clinical practice guidelines to support decision making. The purpose of this systematic review and narrative synthesis was to evaluate the evidentiary bases of recommendations made by stroke clinical practice guidelines regarding the thickened liquids intervention.

**Methods:**

A systematic review was conducted on stroke clinical guidelines retrieved via searches conducted across a range of databases including Academic Search Complete, CINAHL, MEDLINE, and the Cochrane Library as well as through association websites. Guidelines were eligible for inclusion if they focused on adult stroke populations, made recommendations relating to the thickened liquid intervention and were published between January 2010 and December 2018. Four independent reviewers rated methodological quality using the AGREE‐II instrument. Intervention recommendations were extracted and analysed using the Criteria for Levels of Evidence Reported from the Canadian Stroke Best Practice Recommendations and a novel framework examining the appropriateness of the supporting evidence.

**Results:**

Thirteen clinical guidelines were included in the review. Methodological quality was variable with seven rating as good‐excellent overall. Thirty recommendations regarding the intervention were extracted. Of these, 16 recommendations were classed as a recommendation to use the treatment and all guidelines made this recommendation. Much of the evidence used to scaffold recommendations did not directly support the intervention.

**Conclusions:**

Despite the limited evidence base for the thickened liquid intervention, there was consensus among stroke guidelines in recommending it. This is despite limited empirical support. Furthermore, much of the evidence used to support recommendations was not appropriate, suggesting less than satisfactory evidence‐based practices in formulating recommendations. In this case, clinical guidelines may not be reliable decision‐support tools for facilitating clinical decision making.

**Abbreviations**
AGREE‐II: The Appraisal of Guidelines for Research and Evaluation Second Edition
Australia 2017: The Stroke Foundation of Australia CPG
Cameroon 2013: SEEPD Program of the Cameroon Baptist Convention Health Board, the Bamenda Coordinating Centre for Studies in Disability and Rehabilitation (BCCSDR), and ICDR‐Cameroon of the University of Toronto CPG
Canada 2015: Heart and Stroke Foundation (HSF‐C)/Canadian Stroke Best Practices Advisory Committee CPG
Canada 2018: Heart and Stroke Foundation (HSF‐C)/Canadian Stroke Best Practices Advisory Committee CPG
CPGs: clinical practice guidelines
Germany 2013: German Nutrition Society (DGEM) German Society for Neurology, German Geriatric Society CPG
Ireland 2010: Irish Heart Foundation CPG
Philippines 2010: The Stroke Society of the Philippines CPG
PRISMA: Preferred Reporting of Items for Systematic Reviews
PWD: people with dysphagia
TL: thickened liquids
Scotland 2010 #118: Scottish Intercollegiate Network (SIGN) CPG #118
Scotland 2010 #119: Scottish Intercollegiate Network (SIGN) CPG #119
UK 2013: National Institute for Clinical Excellence (NICE) CPG
UK 2016: Royal College of Physicians (RCP) Intercollegiate Stroke Working Party CPG
USA 2010: Veterans Association/Department of Defence (VA/DOD) CPG
USA 2016: American Heart Association (AHA)/American Stroke Association (ASA) CPG

## 1 BACKGROUND

### 1.1 The intervention of thickened liquids

Stroke is a significant cause of oropharyngeal dysphagia which is known to increase the risk of malnutrition, dehydration, aspiration, pneumonia, and mortality.^1^, ^2^ It is estimated that approximately half of stroke patients experience aspiration and one‐third of these develop aspiration pneumonia.^3^ Furthermore, pneumonia is responsible for up to one‐third of post‐stroke deaths.^4^ It is hypothesized that the early detection and treatment of aspiration is important for both individual patients and health care providers, as successful management of aspiration decreases medical complications and related health care expenditures including length of hospital stay.^5^ It is widely accepted that swallowing thin liquids can pose a challenge for people with dysphagia (PWD) including those with dysphagia post‐stroke, because they flow too quickly and can enter the airway below the vocal cords.^6^ Consequently, aspiration is primarily treated by recommending thickened liquids (TL). This is a technique whereby a thickening agent is added to liquids to increase viscosity and slow bolus speed.^7^ It is a common practice worldwide. Approximately 78% of speech and language therapists working with a range of clinical populations and 97% of those working with people with stroke employ the intervention.^8^, ^9^, ^10^ Despite this, the evidence to support TL is not strong.^11^, ^12^, ^13^, ^14^, ^15^ While some recent systematic reviews indicate emerging support,^12^, ^15^ the evidence base remains limited and contradictory with some reviewers even making a weak recommendation against its use.^16^ Decision support tools such as clinical practice guidelines (CPGs) may therefore be especially useful for interventions such as TL.

### 1.2 Clinical practice guidelines

CPGs are “systematically developed statements that assist clinicians to provide appropriate evidence‐based care” (p. 57).^17^ They typically focus on specific conditions such as stroke or dementia and provide evidence based recommendations regarding a range of interventions which are supported by reviews and evaluations of research findings. They aim to bridge the gap between research and practice and have complementary goals of optimizing patient care, decreasing variation in clinical practice, reducing costly, preventable mistakes, and adverse events.^18^, ^19^ Well‐constructed CPGs based on the most up‐to‐date evidence can improve the quality of clinical decision making.^18^ Clinicians tend to view them as key sources of reliable guidance with as many as 78% of health care professionals employing them.^20^ The use of CPGs by clinicians can be interpreted as positive clinical behaviour, illustrating a commitment to evidence based practice and, according to Hurdowar et al, as an indicator of quality of care.^17^ Therefore, establishing the validity of CPG recommendations is important, perhaps especially in cases of interventions which are poorly empirically supported.

Quality CPGs should reflect evidence based practice and thus contain recommendations supported by current research evidence where present. They should also ideally incorporate clinical expertise and patients' perspectives. This triad of evidence may be especially important in cases where the “existing literature is ambiguous or conflicting or where scientific data is lacking or an issue” (p. 6).^21^ The use of practice evidence rather than research‐based evidence to support CPG recommendations is not unusual. A recent review of CPGs for preoperative care for surgical antimicrobial prophylaxis, for example, found that most recommendations were based on clinical experience.^22^ The increased emphasis on person‐centred care has seen a growth in patient evidence being incorporated into decision support tools. A recent systematic review of decision aids by Clifford et al however, suggests this area remains considerably underdeveloped.^23^ In the case of the TL intervention, there are known palatability issues with impacts on independence and quality of life and associated treatment adherence issues.^7^, ^24^ Such patient evidence should be reflected by guidelines when making recommendations.

The evolution of CPGs as sources of reliable clinical information has naturally resulted in research on guideline quality.^25^, ^26^ Recent systematic reviews of stroke CPGs suggest some issues in this area including regarding rigour of development.^16^, ^27^ Navarro‐Puerto et al, for example, reported that the majority of ischaemic stroke CPGs reviewed in their study, received an overall “would not recommend” rating.^27^ There has been considerably less focus on assessing the quality of recommendations made within CPGs—specifically the evidentiary bases of recommendations. Cosgrove et al's examination of recommendations in a major psychiatry CPG showed that fewer than half of the included studies supporting the recommendations met the criteria for high quality. Furthermore, they identified that one‐fifth of the references were not congruent with the recommendations.^28^ As stroke is the leading cause of long‐term adult disability, the quality and content of stroke CPGs may be especially pertinent especially as organized stroke care has been shown to be beneficial and stroke evidence continues to grow rapidly.^17^, ^29^ If Cosgrove et al's findings were to be replicated in stroke CPGs, questions regarding their role in supporting clinical decision making would naturally ensue. Gandhi et al argue that poor‐quality CPGs can be detrimental to clinical practice.^30^ There is a risk that such CPGs may result in the dissemination of inaccurate knowledge, be translated into poor clinical practice, result in waste of resources, be potentially harmful to patients,^19^, ^30^ and contrary to the overall aims of evidence‐based practice generally.

### 1.3 Study aims

The purpose of this systematic review and narrative synthesis is to evaluate the evidentiary bases of stroke CPG recommendations regarding the TL intervention.

## 2 METHOD

There are no specific standardized reporting guidelines relating to systematic reviews of CPGs. To this end, relevant sections of the Preferred Reporting of Items for Systematic Reviews (PRISMA) guidelines^31^ were referenced to standardize the conduct and reporting of the review. The protocol for this review has not been published elsewhere.

### 2.1 Identification of CPGs

A search strategy was developed to identify all stroke CPGs that were published between January 2010 and December 2018. For the purposes of this study, stroke CPGs were defined as CPGs focused primarily on the condition of stroke in both acute and chronic phases and which made recommendations for the rehabilitation of the stroke patient. The search strategies were based on the processes recommended by Moher et al (Appendix S1).^31^ Databases, stroke association websites, and guideline websites were the main sources searched (Appendix S2). Hand‐searching of references of included CPGs was also conducted. Where further information was required and not available in the main document, the main author was contacted.

A sample search strategy for Scopus is presented with the main search terms and related terms (Appendix S3). Core search terms were stroke, guidelines, and TL. Inclusion criteria were as follows:

- CPGs focused on the rehabilitation of stroke populations;
- General stroke CPGs which are not intervention specific (eg, bolus modification) or discipline specific (ie, produced by individual allied health groups);
- CPGs making recommendations relating to the intervention of TL for the management of dysphagia in adults (>18 years) post‐stroke;
- CPGs produced between January 2010 and December 2018;
- Most recent versions of CPGs; and
- Published in English.

CPGs should be reviewed or updated regularly, ideally within 3 to 5 years.^32^, ^33^, ^34^ This was extended in our study to allow for the time required to produce and disseminate them by organizations. Titles and abstracts and guidelines were reviewed by the main author (A.M.) and those that did not meet the criteria were excluded. The remaining publications were reviewed independently by three reviewers (A.M., M.K., C.R.) and consensus regarding inclusion reached on discussion.

### 2.2 Data extraction and analysis

#### 2.2.1 CPG quality

The methodological quality of the included CPGs was evaluated using The Appraisal of Guidelines for Research and Evaluation Second Edition (AGREE‐II) tool.^35^ The AGREE‐II has been established internationally as valid and reliable for quality assessment of CPGs.^36^, ^37^ To facilitate accurate reporting, the organization websites of included CPGs were visited to retrieve relevant documentation, for example, evidence tables, working group declaration of interests, and CPG development frameworks. Where available, these were used in the evaluation process. The AGREE‐II consists of 23 items that are organized into six domains of guideline development: scope and purpose, stakeholder involvement, rigour of development, clarity of presentation, applicability, and editorial independence. Per the AGREE protocol, four reviewers (A.M., D.L., P.B., R.G.) completed the appraisal exercise to minimize variability and optimize validity of results. Each appraiser independently rated the 23 items and met to agree final ratings for items where discrepancies in scoring were evident. Each reviewer provided a rationale for their score and consensus was reached between the four reviewers. Scores across all domains were converted to percentages for each CPG. The AGREE‐II developers advise against calculating an “average” or “overall” score for CPGs representing a combination of the six AGREE‐II domains as each domain is considered more individually informative to the user. Thus, a method previously applied by other authors to determine “high‐quality” was also used, where CPGs achieved a standardized score of ≥50% on all six domains.^38^

#### 2.2.2 Data extraction and evidence analysis

The AGREE‐II does not assess the quality of the CPG content including the evidentiary bases of recommendations. Thus, the extraction and evaluation of TL specific recommendations were performed across a number of domains. These included the specific recommendations, the evidence supporting the recommendations, grading of the evidence based on the Criteria for Levels of Evidence Reported (CLER) in the Canadian Stroke Best Practice Recommendations^39^ (with additional categories), and an examination of the appropriateness of the cited evidence. The details of this framework are shown in Appendix S4. Four reviewers (A.M., D.L., M.K., C.R.) extracted the data, compared the extractions to ensure comprehensiveness, evaluated the extracted data, and agreed the class of recommendation, classification of evidence, and evaluation of included evidence. Results from the AGREE‐II tool are reported as total domain scores in Table 2.

## 3 RESULTS

### 3.1 Included CPGs

**FIGURE 1:**
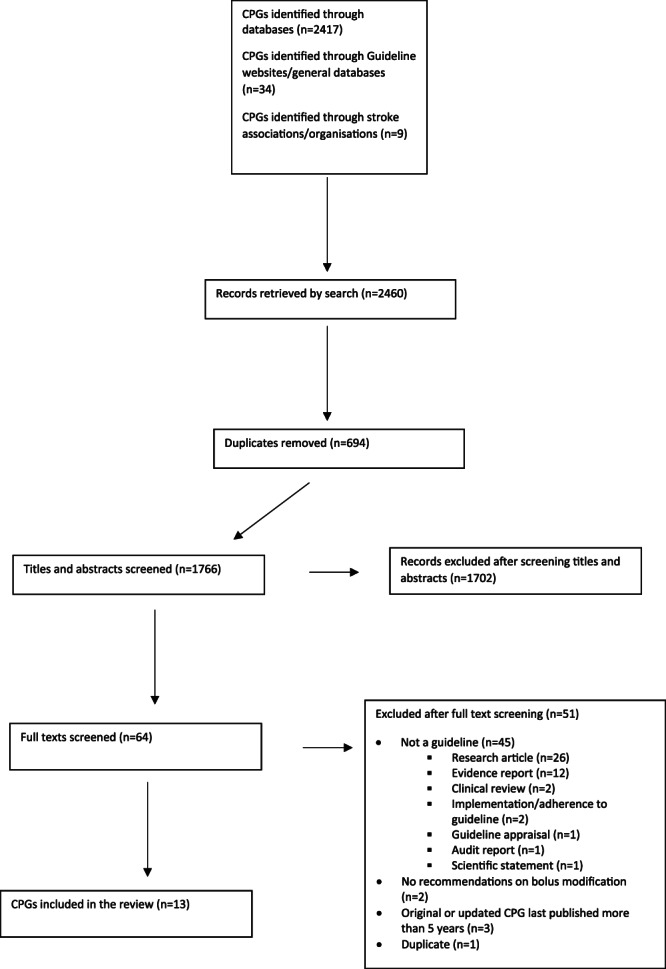
Preferred Reporting of Items for Systematic Reviews (PRISMA) flowchart. To demonstrate the process and results of the literature search, a PRISMA flowchart was utilized and the results are presented. The flow diagram depicts the flow of information through the different phases of the systematic review. It maps out the number of records identified, included and excluded, and the reasons for exclusions. The total number of included papers is outlined in the last box

A total of 2460 documents were initially retrieved from searches. Hand and Google searching retrieved no additional documents. Following removal of duplicates and initial screening of titles and abstracts, 64 documents were considered for full screening. The reviewers independently applied the inclusion criteria to the 64 documents and 13 CPGs were agreed upon for final inclusion (Figure 1) representing six Northern and Continental European (Germany, Ireland, Scotland, United Kingdom), two Australasian (Australia, Philippines), one African (Cameroon), and four North American (Canada, United States) countries/provinces. The CPG selection process is summarized in a PRISMA flow diagram (Figure 1).

The included CPGs (Table 1) were published between 2010 and 2018 and are referenced in text by their country of origin and date of publication. CPGs with the same countries and dates were given added identification information (eg, Scotland 2010 #119). Six CPGs were initially sourced through bibliographic databases (Germany 2013, USA 2010, Canada 2015, Canada 2018, Cameroon 2013, USA 2016) and the remaining sourced through stroke organization and guideline database websites. For most of those retrieved via bibliographic searches, full copies of guidelines and supporting documentation were obtained via the developers' websites. One CPG (Germany 2013) was primarily evaluated using the journal publication as its authors advised that the obtained CPG was the only English version available.

**TABLE 1:** Included stroke CPGs

CPG	Developing organization	Year	Funding/support source
Australia 2017^40^	Stroke Foundation of Australia (SFA)	2017	Australian Government, Department of Health and Ageing Disclaimer that final recommendations not influenced by funding body
Cameroon 2013^41^, ^42^	SEEPD Program of the Cameroon Baptist Convention Health Board, the Bamenda Coordinating Centre for Studies in Disability and Rehabilitation (BCCSDR), and ICDR‐Cameroon of the University of Toronto	2013	Not clearly stated
Canada 2015^43^	Heart and Stroke Foundation (HSF‐C)/Canadian Stroke Best Practices Advisory Committee	2015	Canadian Stroke Network and the Heart and Stroke Foundation
Canada 2018^44^	Heart and Stroke Foundation (HSF‐C)/Canadian Stroke Best Practices Advisory Committee Acute Inpatient Stroke Care Writing Group	2018	Canadian Stroke Network and the Heart and Stroke Foundation
Germany 2013^45^	German Nutrition Society (DGEM) German Society for Neurology, German Geriatric Society	2013	German Nutrition Society (DGEM), German Society for Neurology, German Geriatric Society
Ireland 2010^46^	Irish Heart Foundation (IHA)	2010	MSD Ireland
Philippines 2010^47^	The Stroke Society of the Philippines (SSP)	2010	Not clearly stated
Scotland 2010 #118^48^	Scottish Intercollegiate Network (SIGN)	2010	NHS Quality Improvement Scotland
Scotland 2010 #119^49^	Scottish Intercollegiate Network (SIGN)	2010	NHS Quality Improvement Scotland
UK 2013^50^	National Institute for Clinical Excellence (NICE)	2013	National Institute for Clinical Excellence (NICE)
UK 2016^29^	Royal College of Physicians (RCP) Intercollegiate Stroke Working Party	2016	Royal College of Physicians Clinical Effectiveness and Evaluation Unit
USA 2010^51^	Veterans Association/Department of Defence (VA/DOD)	2010	Office of Quality and Performance VA and Quality Management Division US Army
USA 2016^52^	American Heart Association (AHA)/American Stroke Association (ASA)	2016	The American Heart Association/American Stroke Association

### 3.2 Quality of CPGS

The CPGs generally scored well, with 7/13 scoring above the >50% quality threshold in all six domains. The others tended to score variably across domains with one (Philippines 2010) scoring below this threshold on all domains. Four of the included CPGs (Australia 2017, Canada 2015, UK 2013, UK 2016) scored >70% on all domains. Thus, over half the included CPGs can be considered high‐quality CPGs and nearly a third excellent quality. For all CPGs, the highest scoring domains (aggregated across all CPGs) were Domain 1: scope and purpose (85%) and Domain 4: clarity of presentation (81.8%). CPGs performing better overall tended to achieve higher scores for all domains including, for example, Domain 2 (stakeholder involvement) and Domain 6 (editorial independence). On the basis of AGREE‐II evaluations, most CPGs would be recommended for use (Table 2).

**TABLE 2:** AGREE‐II domain scores

		Domain 1	Domain 2	Domain 3	Domain 4	Domain 5	Domain 6
CPG	%	Scope and purpose	Stakeholder involvement	Rigour of development	Clarity of presentation	Applicability	Editorial independence
Australia 2017	Domain score	83	97	183	79	102	53
	%	98.6	84.4	92.3	93.1	89.6	93.8
Cameroon 2013	Domain score	74	54	90	55	74	17
	%	86.1	39.6	36.9	59.7	60.4	18.8
Canada 2015	Domain score	77	103	175	74	87	54
	%	90.1	90.6	87.5	86.1	74.0	95.8
Canada 2018	Domain score	76	87	136	74	85	52
	%	88.9	74	64.3	86.1	71.9	91.7
Germany 2013	Domain score	54	48	125	72	38	29
	%	58.3	33.3	57.5	83.3	22.9	43.8
Ireland 2010	Domain score	62	39	53	53	45	28
	%	69.4	24	14.9	56.9	30.2	41.7
Philippines 2010	Domain score	39	42	62	44	38	8
	%	37.5	27.1	20.2	44.4	22.9	0
Scotland 2010 #118	Domain score	80	97	167	77	92	34
	%	94.4	84.4	82.7	90.3	79.2	54.2
Scotland 2010 #119	Domain score	75	100	167	66	67	46
	%	87.5	87.5	82.7	75.0	53.1	79.2
UK 2013	Domain score	83	105	190	78	101	48
	%	98.6	92.7	96.4	91.7	88.5	83.3
UK 2016	Domain score	84	98	187	80	104	56
	%	100	85.4	94.6	94.4	91.7	100
USA 2010	Domain score	65	63	137	70	77	20
	%	90.3	49.0	64.9	80.6	63.5	25.0
USA 2016	Domain score	68	52	154	61	72	45
	%	94.4	37.5	75	68.1	58.3	77.1
All	Overall	1000	1086	2006	962	1088	528
	Domain scores	85.0	69.3	73.1	81.8	69.4	67.4

### 3.3 Extraction exercise regarding prescription recommendations for TL

1. *Recommendations*: 30 recommendations were extracted from the included CPGs (Table 3). Of these, 16 recommendations can be classed as a direct recommendation to use the TL treatment and all CPGs made this recommendation. However, only a handful of CPGs (Cameroon 2013, Germany 2013, UK 2016) made an unequivocal recommendation, with most TL recommendations made in the context of offering a suite of interventions for dysphagia. Other recommendations reflected monitoring of and supporting the intervention during implementation including referral to dietitians, staff training, and monitoring of hydration and oral hygiene.
2. *Type of evidence supporting TL recommendations*: The evidence cited to support recommendations is given in Tables 3 and 4. Seven recommendations were not clearly sourced (the authors were unable to identify the pertaining evidence) and two related to consistency descriptor frameworks. Of the remaining, 11 (36.6%) were supported by studies of strong design based on the CLER framework^39^ and one was based on level 2 evidence. Seven recommendations (23%) used information retrieved from other CPGs to support TL recommendations and six (20%) employed consensus. For Recommendation A (recommendation to use), moderate‐strong research evidence emerged as the primary evidentiary source. For Recommendation B (monitoring and implementation of TL) varied sourcing of evidence was evident.
3. *Appropriateness of cited evidence supporting TL recommendations*: A number of key studies were consistently referenced to support TL recommendations with Geeganage et al and Carnaby et al primary among them.^13^, ^59^ While some studies extracted were mentioned explicitly in CPGs, others were embedded in other sources such as references to other clinical guidelines or evidence summaries. For example, Scotland 2010 #119 used the Agency for Healthcare Research and Quality (AHRQ) Evidence Report Summary.^68^ Use of evidence to support the intervention of TL as part of a multicomponent programme tended to be appropriately cited. Apart from this, there was a mismatch between recommendations and evidence, with inappropriate evidence being used in a number of places. Inappropriateness is measured in various ways including use of non‐stroke evidence, evidence not pertaining to the intervention in question, and evidence not providing actual support for the intervention. A commentary is provided on the validity of the specific research sources cited in Table 5.

**TABLE 3:** Thickened liquids recommendations and supporting evidence

Guideline	Extracted recommendation	Class of recommendation	Main evidence cited	Level of evidence used in CPG
Australia 2017: Stroke Foundation of Australia	For stroke survivors with swallowing difficulties, behavioural approaches such as swallowing exercises, environmental modifications, safe swallowing advice, and appropriate dietary modifications should be used early (Chap 3:7/86).	A	Geeganage et al 2012^13^ Bakhtiyari et al 2015^53^	1b (moderate)
	Where stroke patients require modified texture foods and thickened fluids, these should be prescribed using nationally agreed descriptors (Chap 3:86).	B	Cichero et al 2017^54^	Consistency descriptor documents
	Patients with dysphagia on texture‐modified diets and/or fluids should have their intake and tolerance to the modified diet monitored regularly due to the increased risk of malnutrition and dehydration (Chap 3:86).	B	“Practice point”	3 (consensus)
Cameroon 2013: various	Patients with stroke presenting with features indicating dysphagia or pulmonary aspiration should receive a full clinical assessment of their swallowing ability by an appropriately trained specialist who should advise on safety of swallowing ability and consistency of diet and fluids (Rec 64).	A	Bayley et al 2006^55^ Lindsay et al 2010^56^ SCORE 2007^57^	Other guidelines
	The management programme should include compensatory techniques (such as texture modifications and swallowing postures) and rehabilitative techniques (Rec 64).	A	Not clearly stated	Not stated
Canada 2015: Heart and Stroke Foundation	Restorative swallowing therapy and/or compensatory techniques to optimize the efficiency and safety of the swallow, with reassessment as required, should be considered for dysphagia therapy: compensatory techniques may address posture, sensory input with bolus, volitional control, texture modification, and a rigorous programme of oral hygiene (7.1.iv.b).	A	Geeganage et al 2012^13^ Carnaby et al 2006^58^ DePippo et al 1994^59^ SIGN#118 Guidelines^48^ USA 2010 Guidelines^51^ Australia 2010 Guidelines^60^ UK RCP Guidelines 2012^61^	1a (strong) Other guidelines
	Stroke patients with suspected nutritional concerns, hydration deficits, dysphagia or other comorbidities that may affect nutrition (such as diabetes) should be referred to a dietitian for recommendations to meet nutrient and fluid needs orally while supporting alterations in food texture and fluid consistency (7.2.ii.a).	B	Not clearly stated	Not stated
Canada 2018: Heart and Stroke Foundation	Stroke patients with suspected nutritional concerns, hydration deficits, dysphagia, or other comorbidities that may affect nutrition (such as diabetes) should be referred to a dietitian for recommendations: a. to meet nutrient and fluid needs orally while supporting alterations in food texture and fluid consistency recommended by a speech‐language pathologist or other trained professional (9.6.iv).	B	Geeganage et al 2012^13^ Carnaby et al 2006^58^ SIGN#118 Guidelines^48^	1a (strong)
	Guideline also refers reader to Recommendation 7.1.iv.b from Canada 2015‐Rehab above.	A	USA 2010 Guidelines^58^ Australia 2010 Guidelines^60^ UK RCP Guidelines 2012^61^	Other guidelines
Germany 2013: German Nutrition Society	After assessment of the swallowing act a texture modified diet and thickened fluids of a safe texture should be given to patients (Recommendation 30).	A	Clinical consensus point	3 (consensus)
	A dietician should be consulted and nutrition support should be initiated in cases of insufficient intake over a prolonged period of time (Recommendation 31).	B	Not clearly stated	Not stated
Ireland 2010: Irish Heart Foundation	Every patient who requires food or fluid of a modified consistency should be referred to a dietitian. Fluid balance should be monitored carefully when modified consistency drinks and enteral input are given (p. 63).	B	SIGN Guideline No 78 (2004)^62^	Other guidelines
	Advice on diet modification and compensatory techniques should be given following full swallowing assessment (p. 68).	A	Clinical opinion	3 (consensus)
	Patients who are nil by mouth or are on a modified diet should continue to receive clinically essential medication (p. 67).	B	Clinical opinion	3 (consensus)
Philippines 2011 5th revised edition	Dietary modification and compensatory techniques should be taught to patients who are assessed to be able to feed orally safely (E).	A	Not clearly stated	Not stated
	Patients with dysphagia should have an oropharyngeal swallowing rehabilitation programme that includes restorative exercises in addition to compensatory techniques and diet modification (E).	A	Not clearly stated	Not stated
Scotland 2010 #118 SIGN	Patients with dysphagia should have an oropharyngeal swallowing rehabilitation programme that includes restorative exercises in addition to compensatory techniques and diet modification (p. 28).	A	Carnaby et al 2006^58^	1b (moderate)
Scotland 2010 #119: SIGN	Advice on diet modification and compensatory techniques should be given following full swallowing assessment (p. 12).	A	AHRQ Evidence Report Summary 1999^63^ Cook and Kahrilas 1999 ^64^ Logemann et al 1998^65^ Elmstahl et al 1999^66^ Huckabee and Cannito 1999^67^ Klor et al 1999^68^ Rosenbek et al 1996^69^	1a (strong)
	Staff, carers, and patients should be trained in feeding techniques. This training should include: modifications of positioning and diet (7.3).	B	Ramritu et al 2000^70^	1b (moderate)
UK 2013: NICE	Offer swallowing therapy at least three times a week to people with dysphagia after stroke who are able to participate, for as long as they continue to make functional gains. Swallowing therapy could include compensatory strategies, exercises and postural advice (11.1.4 (58)).	A	Carnaby et al 2006 ^58^ Guideline development group consensus	1b (moderate) 3 (consensus)
	Health care professionals with relevant skills and training in the diagnosis, assessment, and management of swallowing disorders should regularly monitor and reassess people with dysphagia after stroke who are having modified food and liquid until they are stable (11.1.4 (60)).	B	NICE Clinical guideline 32^71^ Guideline development group consensus	3 (consensus) Other guidelines
	People who are having thickened food may need assistance with oral hygiene and this should be monitored (11.1.4 (61)).	B	Not clearly stated	Not stated
UK 2016: RCP	Patients with acute stroke who are at risk of malnutrition or who require tube feeding or dietary modification should be referred to a dietitian for specialist nutritional assessment, advice and monitoring (4.7.1.D.).	B	NICE Clinical Guideline 68^72^	Other guidelines
	People with stroke who require food or fluid of a modified consistency should: have the texture of modified food or fluids prescribed using nationally agreed descriptors (4.7.1.G./4.16.1.G)	B	RCSLT and BDA 2003^73^ National Patient Safety Agency 2011^74^	Consistency descriptor documents
	Until a safe swallowing method is established, people with swallowing difficulty after acute stroke should be immediately considered for alternative fluids (4.16.1.B).	A	NICE Clinical Guidelines 32 and 68^71^, ^72^ Geeganage et al 2012^13^	1b (moderate)
	People with stroke with suspected aspiration or who require tube feeding or dietary modification should be considered for instrumental assessment (4.16.1. D).	B	Kertscher et al 2014^75^ Wilson and Howe 2012^76^ Bax et al 2014^77^	2 (limited)
	People with swallowing difficulty after stroke should be considered for swallowing rehabilitation by a specialist in dysphagia management. This should include texture modification of food and/or fluids (4.16.1.F).	A	NICE Clinical Guidelines 32 and 68^71^, ^72^ Geegenage et al 2012^13^ Foley et al 2008^78^ Speyer et al 2010^79^ Rofes et al 2013^80^	1a (strong) Other guidelines
USA 2010: VA/Dept. of Defence	Patients with persistent dysphagia should be offered an individualized treatment programme guided by a dynamic instrumental swallowing assessment. The treatment programme may include modification of food texture and fluids to address swallowing on an individual basis (9.2.a).	A	Foley et al 2008^78^ Bath et al 1999^81^ Elmstahl et al 1999^66^ EBSRS 2009 12th ed.^39^	1a (strong) Other guidelines
	Patients with chronic oropharyngeal dysphagia should be seen for regular reassessment to ensure effectiveness and appropriateness of long‐standing diet, continued need for compensations, and/or modification of rehabilitative techniques (9.2.e).	B	Not clearly stated	Not stated
USA 2016: American Heart Association	Behavioural interventions (including “swallowing exercises, environmental modifications, safe swallowing advice, and appropriate dietary modifications) may be considered as a component of dysphagia treatment (pe21).	A	Geeganage et al 2012^13^ Ashford et al 2009^82^	1a (strong)

**TABLE 4:** Bases of thickened liquid recommendations

Recommendation	CPG	Other guidelines	Research evidence	Clinical opinion/consensus	Not stated	Consistency descriptor documents
A. Recommend use of TL	Australia 2017		✓			
	Cameroon 2013	✓				
	Canada 2015	✓	✓			
	Germany 2013			✓		
	Ireland 2010			✓		
	Philippines 2011				✓	
	Scotland 2010 #118		✓			
	Scotland 2010 #119		✓			
	UK 2013		✓			
	UK 2016	✓	✓			
	USA 2010	✓	✓			
	USA 2016		✓			
B. Monitoring and implementation of TL	Australia 2017			✓		✓
	Canada 2015	✓			✓	
	Canada 2018	✓	✓			
	Germany 2013				✓	
	Ireland 2010	✓				
	Scotland 2010 #119		✓			
	UK 2013	✓		✓	✓	✓
	UK 2016	✓	✓			✓
	USA 2010				✓	
Total sources		9	11	4	5	3
% of all sourcing		28.1%	34.4%	12.5%	15.6%	9.4%

**TABLE 5:** Appropriateness of main evidence

Study	Comment on evidence	Does study examine TL intervention specifically?	Can effects of TL be isolated?	CPGs using evidence	For which recommendation
Geeganage et al 2012^13^	Systematic review of dysphagia interventions in stroke. Based on one RCT—Garon et al 1997^83^ evaluating hydration in TL and water protocols. No implications for efficacy of TL can be drawn.	N	N	Australia 2017	A
				Canada 2018	B
				Canada 2015	B
				UK 2016	A
				USA 2016	A
Bakhtiyari et al 2015^53^	Randomized clinical trial. Patients allocated to groups based on the timing of initiation of swallowing therapy after the stroke. A range of interventions used including traditional swallowing therapy.	N	N	Australia 2017	A
Singh and Hamdy 2006^84^	Recommendations based on guideline which used this review. Review concludes that while numerous studies have described the changes in swallowing physiology in people with stroke taking TL, none have shown clinical efficacy.	N	N	Cameroon 2013	A
Carnaby et al 2006^58^	Does not specifically examine TL in isolation but as multicomponent intervention. The effectiveness of TL as a treatment cannot be isolated/supported based on these papers.	Y	N	Canada 2018	B
				Canada 2015	A
				Scotland 2010#118	A
				UK 2013	A
				UK 2016	A
DePippo et al 1994^59^	Three graded levels of dysphagia therapist control of diet consistency and reinforcement of compensatory swallowing techniques were provided. No significant difference between the three treatment groups. The effectiveness of TL as a treatment cannot be isolated/supported based on these papers.	Y	N	Canada 2015	A
Cook and Kahrilas 1999^64^	Literature review. Studies included multicomponent programmes (Groher, 1987; DePippo et al 1994; Neumann et al 1993; Neumann et al 1995) retrospective reviews (Kaspairn et al 1989), varied/non‐stroke patients (Martens et al 1990; Neumann et al 1993; Silbergleit et al 1991), non‐significant outcomes (eg, Martens et al 1990) and studies where TL not included (Neumann et al 1995).	Y/N	N	Scotland 2010 #119	A
Logemann et al 1998^65^	Study examining instrumentation to assess swallow function.	N	N	Scotland 2010 #119	A
Elmstahl et al 1999^66^	Observational study reporting on the effects of swallowing techniques on nutritional and anthropometric variables. TL included. Found benefits for multicomponent swallowing therapy.	Y	N	Scotland 2010 #119	A
				USA 2010	A
Huckabee and Cannito 1999^67^	Retrospective review of multicomponent programme with a group of 10 patients with chronic dysphagia subsequent to a single brainstem injury.	Y	N	Scotland 2010 #119	A
Klor et al 1999^68^	Sixteen nursing home patients on PEG feeding post stroke. Non‐randomized study. Multicomponent programme.	Y	N	Scotland 2010 #119	A
Rosenbek et al 1996^69^	Thermal intervention (icing).	N	N	Scotland 2010 #119	A
Ramritu et al 2000^70^	Systematic review on nursing interventions for broad range of paediatric and adult individuals with dysphagia due to neurological impairment. Limited/ not directly applicable research evidence.	N	N	Scotland 2010 #119	B
Kertscher et al 2014^75^	Systematic review of bedside screenings in a range of neurological patients. Variable results.	N	N	UK 2016	B
Wilson and Howe 2012^76^	Cost effectiveness analysis of VFSS. Investigation of dysphagia with instrumental assessments helps to predict outcomes and improve treatment planning.	N	N	UK 2016	B
Bax et al 2014^77^	Retrospective study. Significant findings in favour of FEES and reduced rates of pneumonia and return to standard diet. Negative findings in terms of length of hospital stay and non‐oral feeding	N	N	UK 2016	B
Speyer et al 2010^69^	Systematic review not specific to stroke. Included a range of dysphagia interventions. Difficult to draw strong conclusions about the effectiveness of TL as a treatment from the reviews.	Y	N	UK 2016	A
Foley et al 2008^78^	Systematic review found that general dysphagia therapy programmes are associated with a reduced risk of pneumonia in the acute stage of stroke. Concluded that there was limited/inconclusive evidence.	Y	N	UK 2016	A
				USA 2010	A
Rofes et al 2013^80^	Non‐randomized, non‐intervention study. Mixed population. Assessment of specific commercial product. Positive effects for TL. Commercially funded.	Y	Y	UK 2016	A
Bath et al 1999^81^	Systematic review including multicomponent programmes/multiple interventions/multiple outcomes. Concluded that there was limited/inconclusive evidence.	Y	N	USA 2010	A
Ashford et al 2009^82^	Systematic review regarding behavioural dysphagia interventions (postural interventions swallowing manoeuvers) for a range of neurological disorders. Did not include TL.	N	N	USA 2016	A

## 4 DISCUSSION

This is the first review that examines both the quality of stroke CPGs and the evidentiary bases of recommendations made regarding the TL intervention. While nearly half the CPGs that fitted the inclusion criteria were sourced from bibliographic databases, the remaining were retrieved from professional association and guideline websites and the documentation required to support the exercise was mostly available on their websites. This resonates with the ADAPTE Collaboration commentary that many guideline developers are posting CPGs directly online and not publishing through peer reviewed bibliographic sources.^85^ This approach has both benefits and disadvantages. The advantages include better accessibility to CPGs by both the general public and clinicians as well as flexibility to rapidly update online versions with new evidence. However, unless the processes for developing and updating CPGs are both explicit and rigorous, they may not be subject to the same peer review process as for CPGs published in the literature. In this study, a clear underuse of recent systematic reviews for the intervention was evident for most CPGs, irrespective of whether they were published through association websites or in journals. A number of CPGs (eg, UK 2013) have notably attempted to improve rigour by building in external peer review elements to the guideline development process.

### 4.1 The quality of CPGs

While the quality of stroke CPGs based on the AGREE‐II evaluation was variable, just over half rated as generally good to excellent. Although there is space for improvement, this reflects well on guideline developers attempts to improve process rigour when developing CPGs,^17^, ^27^ and is consistent with Rohde et al's recent systematic review of stroke CPGs and aphasia.^86^ Domain 1 (scope and purpose) and Domain 4 (clarity of presentation) were the highest scoring domains. Furthermore, most CPGs scored well in the Domain 5: rigour of development, the exceptions being Cameroon 2013, Ireland 2010, and Philippines 2010. Domain 5 is fundamental to the integrity and transparency of CPGs and often considered most indicative of CPG quality.^17^ Despite this, there was some variability in scoring for domains both within and between CPGs which suggests ongoing improvements are required. Scores for some domains may reflect a lack of availability and/or explicitness on the part of CPG documentation. In a number of cases, the authors felt unable to credit CPGs with points on the AGREE‐II scoring tool due to a lack of available information. This includes Germany 2013 as the only English material available was the published paper. It is assumed further supporting documentation would be available in its original language.

### 4.2 The evidentiary bases of recommendations

The consensus across all CPGs was that the intervention of TL should be used for people with aspiration subsequent to stroke, either in isolation or as part of a general dysphagia treatment programme. The evidence base pertaining to the intervention of TL is less than robust and therefore a mismatch between evidence and recommendations is apparent. Clinicians who access CPGs may do so due to a lack of time to read, appraise, and synthesize all the available evidence on the topic, leading to a reliance on CPGs to provide evidence‐based recommendations. Therefore, the unequivocal recommendations by the included CPGs to administer TL for PWD post‐stroke are problematic. This was commonly magnified by the lack of editorial commentaries highlighting the poor empirical support. The very nature of summation integral to CPGs may in itself undermine informed clinical decision making. To investigate recommendation sources, the authors of this study had to repeatedly perform searches to identify and retrieve papers not clearly cited. This is not a task busy clinicians can or should have to perform.

There were difficulties with the evidentiary bases of TL recommendations across most CPGs. In many cases the cited studies did not clearly provide evidence of efficacy for the treatment of TL. Where such justification was provided, it was only as part of multi‐component dysphagia programmes. While most recommendations reflected this—either directly by recommending use of TL as part of such a programme or by broad statements reflecting a suite of possible treatments—this evidence does not provide support for the efficacy of the TL treatment. This important caveat was rarely clearly stated. Other supporting evidence did not relate specifically or wholly to the population under scrutiny. Examples include Garon et al which did not specifically examine TL,^83^ and systematic reviews such as Speyer et al which examined a range of clinical populations.^79^ Furthermore, some evidence was used to support recommendations without relevant context or clarity being provided. An example of this was the Foley et al systematic review.^78^ Foley et al plainly summarize that: “there is scant empirical evidence of its medical effectiveness due to a number of factors …making it difficult to establish which component was associated with pulmonary benefit” (p. 262).^78^ Ideally, when evidence is used to support recommendations, a corresponding note on the original authors' conclusions should be made to provide context and ensure clarity in translation.

Two studies in particular were utilized by a number of CPGs. One was the systematic review of Geeganage et al^13^ which was effectively based on a randomized control trial^83^ which evaluated hydration in TL and free water protocols (a different intervention), with limited implications for the efficacy of TL. The other frequently cited paper was Carnaby et al, an RCT which evaluated a multicomponent dysphagia programme and from which the effects of TL cannot be isolated.^58^ Furthermore, the more recently produced GPGs did not tend to include newer systematic reviews^11^, ^12^, ^14^, ^15^ including that of Beck et al who recommended against the use of TL.^16^ While such deficits may in part result from the time required to develop and produce new versions of guidelines, an efficient process for retrieving and including most recent research is warranted to reflect the most up‐to‐date evidence. Martínez García et al noted that such a mechanism is imperative given their findings that CPG recommendations quickly become outdated, with one out of five recommendations being out of date after 3 years.^87^ Protocols for removing outdated CPGs from circulation are therefore also warranted. In many cases, previous versions of the included CPGs were still available and a number of “older” CPGs were still in use.

The employment by guideline developers of other CPGs as sources of evidence and support for recommendations was also noted. This reliance can be understood to be less than ideal, especially in the context of the findings from this study. Furthermore, the range of sources to support recommendations was generally not extensive and did not always reflect the ideal triad of evidence from practice and patient perspectives. Where consensus was employed, this usually reflected consensus within the working group and not a broader representation of individuals directly involved in prescribing and implementing highlighted interventions.^23^ There also tended to be poor consideration of other important factors such as contextual evidence which has been clearly shown to factor in treatment implementation in a number of health disciplines.^88^, ^89^ Furthermore, contemplation of patient and ethics‐based evidence including possible treatment burden and intervention risks and benefits were generally absent. This is especially important for interventions such as TL where there is clear evidence of patient dislike and treatment adherence issues.^7^ It is reasonable to argue that CPG recommendations should be better grounded in a range of information and evidence and frameworks such as the total evidence and knowledge approach^90^ may help in this regard. This may be especially so in the cases of interventions with limited empirical evidence such as TL.

What can be positively inferred is the attempt by most CPGs working groups to systematically review the evidence. Furthermore, some CPGs do provide recommendation rationales. Scotland 2010 #119, for example, notes that despite the sparse available data, it seems “prudent” to include dysphagia‐specific management as part of the standard protocol of stroke management in the acute care setting: “Diet modification has been shown to be effective in specific individuals using video‐fluoroscopy and is standard management of dysphagia following stroke” (p. 12).^49^ It is clear most of the CPGs involved are attempting to make “best” recommendations in the context of limited research evidence. However, the misappropriation of evidence, non‐use of recent evidence, limited use of a range of evidence, and the failure to clearly report the state of the evidence when recommending TL raises questions about the reliability of CPG recommendations. It seems true, as Elwyn et al have noted, that “the reality is that busy clinicians are working with guidelines that represent imperfect knowledge” (p. 2). ^91^ Irrespective, guideline developers have a responsibility to make truly evidence based recommendations, use up‐to‐date evidence, be explicit about the strengths and limitations of the evidence they use to support their recommendations, and use that evidence appropriately.

## 5 RECOMMENDATIONS

### 5.1 For guideline developers

- Guideline developers should make reasonable efforts to employ the best and most recent evidence when making recommendations and CPGs should preface intervention‐specific recommendations with clear summaries of the evidence‐base.
- Supporting evidence should be specific to the intervention being recommended. Furthermore, explicit links between supporting evidence and individual recommendations should be consistently employed to enable readers to assess the evidence and encourage guideline developers to provide targeted evidence for specific recommendations.
- Clear directions and easy access to all supporting documentation are required to enable future researchers and policy makers to fully understand the evidence on which recommendations are being made, including caveats to recommendations.
- A broader range of evidence should be considered in formulating recommendations especially in cases where the intervention evidence is limited. This may include ethical, contextual, and collective patient evidence.
- Guidelines developers should consider the use of expert panels with intervention‐specific expertise when evaluating evidence and making and reviewing recommendations. Where recommendations are based on such evidence, this should be explicitly stated to ensure readers understand the nature of the evidence supporting that recommendation and can act accordingly.
- Care should be taken to ensure that traditional clinical practices are not automatically recommended or assumed to be best practice in the absence of supporting evidence.
- To ensure that publicly available guidelines remain current, processes for updating CPGs need to be explicit and rigorous. For example, UK 2013 CPG authors designed ongoing external peer review elements to their guideline development process. A process for removal or CPGs is also indicated based on the findings of this review.

### 5.2 For individuals and organizations employing CPGs

- Individuals, teams, and organizations employing CPGs should be aware that CPG recommendations may not be wholly evidence‐based and should review the evidence where possible to ensure recommendations are evidence‐based.
- Clinicians may benefit from training in CPG critical appraisal in order to be able to evaluate the supporting evidence used by CPGs. Such investments would maximize the applicability of CPGs as well as increase the number of clinicians who may, in the future, contribute to CPG content themselves.

## 6 STRENGTHS AND LIMITATIONS

Systematic methods were used to identify, select, appraise, and synthesize the findings from the included CPGs. The PRISMA standardized reporting guidelines were referenced to illustrate the flow of studies in the review. The interdisciplinary review team reflected a mix of academics and clinicians. This is first review to examine the evidence underpinning recommendations in stroke CPGs.

Some limitations are evident. Comparatively, this review includes a small sample size. Possible explanations for this include the use of TL as a core term in the search string and the timeframe imposed to maximize retrieval of the most current versions of CPGs. Additionally, although it is advised that CPGs are updated every 3 to 5 years, this was not the case for a number of CPGs in the review. This means that some included CPGs could not reflect up to date evidence. Furthermore, the inclusion criteria did not capture CPGs published by professional speech and language therapy associations which may reflect deeper discipline‐specific knowledge regarding the research evidence and the intervention itself. This may limit the external validity of the findings. Where possible the evidence referred to in “other guidelines” was also extracted. In some cases this was not possible due to guideline age, lack of access to original documentation or being superseded by a subsequent guideline. Some CPGs, such as Germany 2013, may not have been best reflected in this exercise due to lack of accessibility to guideline development documents.

## 7 CONCLUSION

This study examined the evidentiary bases of recommendations for the intervention of TL for PWD post‐stroke. The quality of the 13 stroke CPGs included in this study was generally good, based on tools examining the development of those guidelines. A discrepancy was highlighted between quality rating tools for guidelines and the narrative evaluation of the evidence underpinning guideline recommendations. Despite the limited evidence base for the TL intervention, there was consensus evident among CPGs in recommending it. Furthermore, much of the evidence used to support recommendations was inappropriate, suggesting less than satisfactory evidence‐based practices in formulating recommendations. CPGs may therefore not be the most reliable decision support tools with which to facilitate evidence based clinical decision making and clinicians may be better served by guidelines that target specific interventions rather than broad‐based instruments. Furthermore, if these findings are reflected for other interventions and in other CPGs, questions should be raised about the value and reliability of guidelines generally.

## CONFLICT OF INTEREST

The authors declare no conflicts of interest.

## Supporting information


**Data S1**. PRISMA checklist. The PRISMA checklist is an evidence‐based minimum set of items for reporting in systematic reviews and meta‐analyses. It uses explicit, systematic methods that are selected to minimize bias, thus providing reliable findings from which conclusions can be drawn and decisions made. The 27 checklist items pertain to the content of a systematic review and Appendix S1 details the completed checklist for this review.

**Data S2**. Main sources searched for CPGs. This table identified the databased employed in the search and included stroke association and guideline websites.

**Data S3**. Search terms. Appendix S3 highlight the search terms employed in this exercise.

**Data S4** Extraction Framework. This table provides details of the extraction framework which was developed to support the narrative synthesis.
