# Supplementary material for: Do stroke clinical practice guideline recommendations for the intervention of thickened liquids for aspiration support evidence based decision making? A systematic review and narrative synthesis
Source: J Eval Clin Pract. 2020 Feb 21;26(6):1744–60. doi: 10.1111/jep.13372 (PMC7687236; doi:10.1111/jep.13372)
Supplement: Supplementary file 2 — Data S2. Main sources searched for CPGs. This table identified the databased employed in the search and included stroke association and guideline websites. [file JEP-26-1744-s002.docx]

Appendix 2 Main sources searched for CPGs

| **Format** | **Examples** | **Rationale** |
| --- | --- | --- |
| Bibliographic electronic databases | Academic Search Complete, CINAHL, Biomedical Reference Collection, MEDLINE, Omni File, Social Sciences, General Science, PsycINFO, Allied and Complementary Medicine Database, PsycARTICLES, Scopus, Cochrane Library, PubMed, Embase. | Retrieval of published peer reviewed CPGs |
| Stroke association websites and guideline databases | The Irish Heart Foundation, Royal College of Physicians UK, National Institute for Healthcare and Excellence, Scottish Intercollegiate Guideline Network, European Stroke Organisation, American Heart Foundation and American Stroke Association, Canadian Heart and Stroke Foundation, National Stroke Foundation Australia and the Stroke Foundation of New Zealand | CPGs produced by speciality associations may be published directly online and not through bibliographic databases |
| Accessible Guideline database websites | National Guidelines Clearing House, Guidelines International Network, Canadian Medical Association, Registered Nurses Association of Ontario, Guidelines International Network, Institute for Clinical Systems Improvement, Ministry of Health New Zealand, and the Canadian Agency for Drugs and Technologies in Health | Guideline databases and websites which produce / itemise evidence-based clinical guidelines and where membership is not required to access content. |
| Hand searching (snowballing) | Included CPG reference lists | To identify CPGs not retrieved from other searches. |
| General internet search | Google | To identify CPGs not retrieved from other searches. |
