# Supplementary material for: Do stroke clinical practice guideline recommendations for the intervention of thickened liquids for aspiration support evidence based decision making? A systematic review and narrative synthesis
Source: J Eval Clin Pract. 2020 Feb 21;26(6):1744–60. doi: 10.1111/jep.13372 (PMC7687236; doi:10.1111/jep.13372)
Supplement: Supplementary file 3 — Data S3. Search terms. Appendix S3 highlight the search terms employed in this exercise. [file JEP-26-1744-s003.docx]

Appendix 3 Search terms

|  | stroke |
| --- | --- |
|  | CVA |
|  | cerebral accident |
|  | cerebrovascular accident |
|  | OR/1-4 |
|  | guideline* |
|  | clinical guideline* |
|  | practice guideline* |
|  | consensus |
|  | expert consensus |
|  | opinion* |
|  | expert opinion* |
|  | expert testimon* |
|  | clinical consensus |
|  | clinical opinion |
|  | consensus document |
|  | OR/6-16 |
|  | deglutition disorder* |
|  | dysphagia |
|  | swallow* disorder* |
|  | swallow* difficult* |
|  | OR/18-21 |
|  | thick* liquid |
|  | thick* fluid |
|  | thick* diet |
|  | thick* beverage |
|  | thick* drink |
|  | thick* bolus |
|  | modif* liquid |
|  | modif* fluid |
|  | modif* diet |
|  | modif* beverage |
|  | modif* drink |
|  | modif* bolus |
|  | liquid modif* |
|  | fluid modif* |
|  | diet modif* |
|  | beverage modif* |
|  | drink modif* |
|  | bolus modif* |
|  | liquid viscos* |
|  | fluid viscos* |
|  | diet viscos* |
|  | beverage viscos |
|  | drink viscos* |
|  | bolus viscos* |
|  | OR/23-46 |
|  | AND/ 5,17, 22, 47 |
|  | parkinson* disease |
|  | PD |
|  | traumatic brain injury |
|  | TBI |
|  | acquired brain injury |
|  | ABI |
|  | pediatric* |
|  | paediatric* |
|  | child |
|  | adolescen* |
|  | OR/49-58 |
|  | NOT/59 |
