# Supplementary material for: Do stroke clinical practice guideline recommendations for the intervention of thickened liquids for aspiration support evidence based decision making? A systematic review and narrative synthesis
Source: J Eval Clin Pract. 2020 Feb 21;26(6):1744–60. doi: 10.1111/jep.13372 (PMC7687236; doi:10.1111/jep.13372)
Supplement: Supplementary file 4 — Data S4 Extraction Framework. This table provides details of the extraction framework which was developed to support the narrative synthesis. [file JEP-26-1744-s004.docx]

Appendix 4 Extraction framework

| **Extraction element** | **Description** | **Components** | **Table no.** |
| --- | --- | --- | --- |
| Recommendations | Inductive classification of recommendations based on review of CPGs | *A* - Recommendation to use the TL intervention.  *B* - Recommendation relating to the monitoring or implementation of TL. | 4 |
| Evidence cited to support recommendation | Listing of evidentiary sources | Named sources | 5 |
|  | Classification of evidence based on Criteria for Levels of Evidence Reported in the Canadian Stroke Best Practice Recommendations- CLER^39^ | *Level 1a (Strong) -* Findings supported by results of two or more RCTs of at least “fair” quality.  *Level 1b (Moderate) -* Findings supported by a single RCT of a least “fair” quality.  *Level 2 (Limited) -* Findings supported by at least one non-experimental study (non-RCT, cohort studies, etc.).  *Level 3 (Consensus) -* In the absence of evidence agreement by a group of experts on the appropriate treatment course.  *Level 4 (Conflicting****)*** *-* Disagreement between the findings of at least two RCTs. | 5 |
|  | Two additional classifications were added in response to information retrieved during exercise | *Other guidelines* - A number of CPGs referred to other CPGs as the bases for their own recommendations. Where possible this evidence was also extracted.  *Consistency descriptors* *document* - Not research evidence but frameworks regarding the terminology of different TL consistencies. | 5 |
| Applicability of cited evidence | Analysis of evidence to determine suitability of evidence to support TL recommendation | *Description of specific evidence* – e.g. type and content of study  *Did study examine TL intervention specifically?* - Identification as to whether study specifically examined TL intervention.  *Can effects of TL be isolated?* **-** did study provide direct support for TL intervention in isolation.  *CPGs using specific evidence* – Identification of which CPGs used which evidence.  *For which recommendations was evidence used -* Identification of which type of recommendations evidence was used to support. | 6 |
